# The impact of first birth obstetric anal sphincter injury on the subsequent birth: a population-based linkage study

**DOI:** 10.1186/s12884-015-0469-4

**Published:** 2015-02-13

**Authors:** Amanda J Ampt, Christine L Roberts, Jonathan M Morris, Jane B Ford

**Affiliations:** Clinical and Population Perinatal Health Research, Kolling Institute, University of Sydney, Royal North Shore Hospital, Building 52, St Leonards, NSW 2065 Australia

**Keywords:** Obstetric anal sphincter injury, Recurrence, Population, Third/fourth degree tear

## Abstract

**Background:**

With rising obstetric anal sphincter injury (OASI) rates, the number of women at risk of OASI recurrence is in turn increasing. Decisions regarding mode of subsequent birth following an OASI are complex, and depend on a variety of factors. We sought to identify the risk factors for OASI recurrence from first and subsequent births, and to investigate the effect of OASI birth factors on planned caesarean for the second birth.

**Methods:**

Using two linked population datasets from New South Wales, Australia, we selected women giving birth between 2001 and 2011 with a first birth OASI and a subsequent birth. Multivariable logistic regression was used to identify the association of first and second birth factors with OASI recurrence, and to determine which factors were associated with a planned pre-labour caesarean at the second birth.

**Results:**

Of 6,380 women with a first birth OASI who proceeded to a subsequent birth, 75.4% had a vaginal second birth, 19.4% a pre-labour caesarean, and 5.2% an intrapartum caesarean. Although the OASI recurrence rate of 5.7% was significantly higher than the first birth OASI rate of 4.5% (p < 0.01), this may not reflect a clinically significant increase. Following adjustment for first and second birth factors, first birth diabetes and second birthweight ≥3.5 kg were associated with increased likelihood of OASI recurrence, while first birthweight ≥4.0 kg and second gestation at 37–38 weeks were associated with decreased likelihood. A fourth degree tear at the first birth was the strongest factor associated with planned caesarean at the second birth, with other factors including epidural, spinal or general anaesthetic, birthweight, gestation, country of birth and maternal age.

**Conclusions:**

Compared with previous reports, the low OASI recurrence rate (approximately one in twenty) may reflect appropriate decision-making about subsequent mode of delivery following first birth OASI. This assertion is supported by evidence of different risk profiles for women who have planned caesareans compared with planned vaginal births.

## Background

Obstetric anal sphincter injuries (OASIs) are recognised as a serious complication of vaginal births, and can result in long term problems including anal incontinence, ongoing perineal pain, dyspareunia and complex psychological issues [1-4]. A recent meta analysis of published risk factors reported primiparity, increased birthweight, median episiotomy and instrumental birth as being associated with OASI [5].

Within the last twenty years reported rates of OASI have varied considerably, from less than 0.6% in Finland [6] to a primiparous rate of 16% within a large US hospital [7]. Despite this variation, there is agreement that OASI rates are rising [6,8-10]. For example, among primiparous women in Australia the rate has increased from 4.1% in 2001 to 5.3% in 2009 [8], with population studies from Scandinavia revealing rate increases of approximately 400% over the past four decades [6]. With a higher proportion of women experiencing a first OASI, the number at risk of a recurrence in a subsequent birth has in turn increased. The reported recurrence rates vary from 2.0% [11] to 13.4% [12], depending on population and study design. Investigation into recurrence risk has generally focussed on factors around the birth subsequent to the OASI, with similar risk factors reported as those for a first OASI [7,11-18].

Not surprisingly, there is general agreement that women feel apprehensive about subsequent births, with some women wishing to delay a further pregnancy [1,4,19]. The risk of recurrence is a major factor in planning the mode of a subsequent birth. In some populations women with a prior OASI are reported to be more likely to have a caesarean for the next birth [13,20]; however a Swedish study reported that very few caesarean sections were performed for this indication [18].

In recognition of the complexities in counselling a woman approaching a second birth following an OASI at her first, we undertook a large population-based study to determine risk factors from first and second births for OASI recurrence. In addition, we examined the effect of first birth factors on planning for either caesarean or vaginal second birth.

## Methods

### Population

The study population consisted of women who sustained an OASI at first birth and proceeded to a subsequent second birth in New South Wales (NSW) between 2001 and 2011. NSW is the most populous state in Australia, and with over 95,000 births occurring in 2011, it contributes to approximately one third of all Australian births [21]. Where a multiple pregnancy occurred, data pertaining to the first born infant were used for analysis.

### Data sources and variables

Data sources consisted of two population-based previously validated data collections: the NSW Perinatal Data Collection (birth data) and the NSW Admitted Patients Data Collection (hospital data). The birth data are a legislated surveillance of demographic characteristics, pregnancy, and maternal and infant outcomes for all births in NSW ≥20 weeks gestation or ≥400 g birthweight, with data recorded by the attending midwife or doctor. The hospital data collection is a census of all admissions to NSW hospitals, with diagnoses and procedures coded from clinical patient records according to the International Classification of Diseases, Australian Modification (ICD-10-AM) and the Australian Classification of Health Interventions (ACHI) [22,23]. The NSW Centre for Health Record Linkage (CHeReL) undertook probabilistic longitudinal linkage of these two datasets using methods previously described [24], with de-identified data provided to researchers.

Obstetric anal sphincter injuries (OASIs) were identified from the hospital data by the ICD-10-AM diagnosis codes: ‘O70.2’ (third degree perineal laceration during delivery) or ‘O70.3’ (fourth degree perineal laceration during delivery), or by the ACHI procedure coding ‘16573-00’ (suture of third or fourth degree tear of the perineum) [22,23]. With a sensitivity of 94.2 and a positive predictive value of 99.7, this combination of codes has been reported as the most reliable indicator for identifying OASIs for NSW population health data [25]. All OASI rates are reported among vaginal births.

Recognised risk factors for OASIs [5,8,16,26-28] that were available in the population data were identified by the most reliable source as reported by previous validation studies [25,29-31]. Birth data were used to identify instrumental birth (forceps or vacuum), gestation, maternal age, infant sex and birthweight, epidural and/or spinal analgesia, induction or augmentation, and year of birth; while hypertension (chronic or pre-eclampsia/eclampsia), diabetes (gestational or diabetes mellitus) were identified exclusively from the hospital data. Asian ethnicity is a recognised risk factor for OASI [8]. As ethnicity is not available in the population data, country of birth as reported in the birth data was used to identify women of Asian background. Episiotomy was identified if reported by either birth or hospital data collections. Neither the birth nor hospital data specify the type of episiotomy, but typical Australian practice is to perform a mediolateral incision according to clinical perception of need.

### Analyses

Firstly, we determined the first birth OASI rate among women having vaginal births who proceeded to a second birth. We then calculated the rates for second vaginal birth, intrapartum and pre-labour caesarean, and OASI recurrence. The OASI first birth rate and OASI recurrence rate were compared using McNemar’s test of paired data.

### Association of first and second birth factors with OASI recurrence

Descriptive analysis was used to determine the distribution of birth factors from the first and second births among women with an OASI recurrence, and among those without. The crude odds ratios (cORs) were calculated. First birth risk factors that were considered included episiotomy, mode of birth, birthweight and analgesia/anaesthesia. As regional or general anaesthetic is recommended for OASI suturing to allow for repair without tension [32,33], a combined analgesia/anaesthesia variable of epidural or spinal or general anaesthetic was created. In addition, diabetes and interpregnancy interval were included as they may influence healing after first OASI. Factors from the second birth were chosen for previously recognised association with OASI and included maternal age, hypertension, gestation, induction or augmentation, epidural analgesia, mode of birth, episiotomy, birthweight, infant sex and year of birth. All potentially predictive birth factors were entered into a multivariable logistic regression model, and adjusted odds ratios (aORs) reported.

### Association of first birth and second pregnancy factors with planned caesarean section for subsequent birth

Using information regarding labour onset, we categorised caesareans as pre-labour (recorded as ‘no labour’) or intrapartum (recorded as ‘spontaneous labour’ or ‘induced’). Women who underwent a pre-labour caesarean were classified as ‘planned caesarean’. Among the intrapartum caesarean group, some women may have been booked for a planned caesarean but commenced spontaneous labour prior to the planned date. These women were also classified as ‘planned caesarean’ if their medical record indicated that labour was not intended. Such indications included not having had an induction nor augmentation, and the reason for caesarean section not related to fetal distress nor failure to progress. All other women who laboured were classified as ‘planned vaginal’, including those from the intrapartum caesarean group who did have an induction or augmentation, or whose reason for caesarean was ‘failure to progress’ or ‘fetal distress’.

The distribution of factors potentially influential in decision-making for mode of second birth was compared between the ‘planned vaginal’ group and the ‘planned caesarean’ group. These factors included country of birth; morbidity (diabetes or hypertension), gestation, induction/augmentation, analgesia/anaesthesia, instrumental birth, episiotomy, third or fourth degree tear, and birthweight at first birth, as well as interpregnancy interval. Second pregnancy factors included maternal age, diabetes, hypertension, gestation, and year of second birth. Multivariable logistic regression was then used to ascertain aORs for factors that may be predictive of planned caesarean.

### Ethics approval

Ethics approval was obtained from the NSW Population and Health Services Research Ethics Committee.

## Results

**Figure 1:**
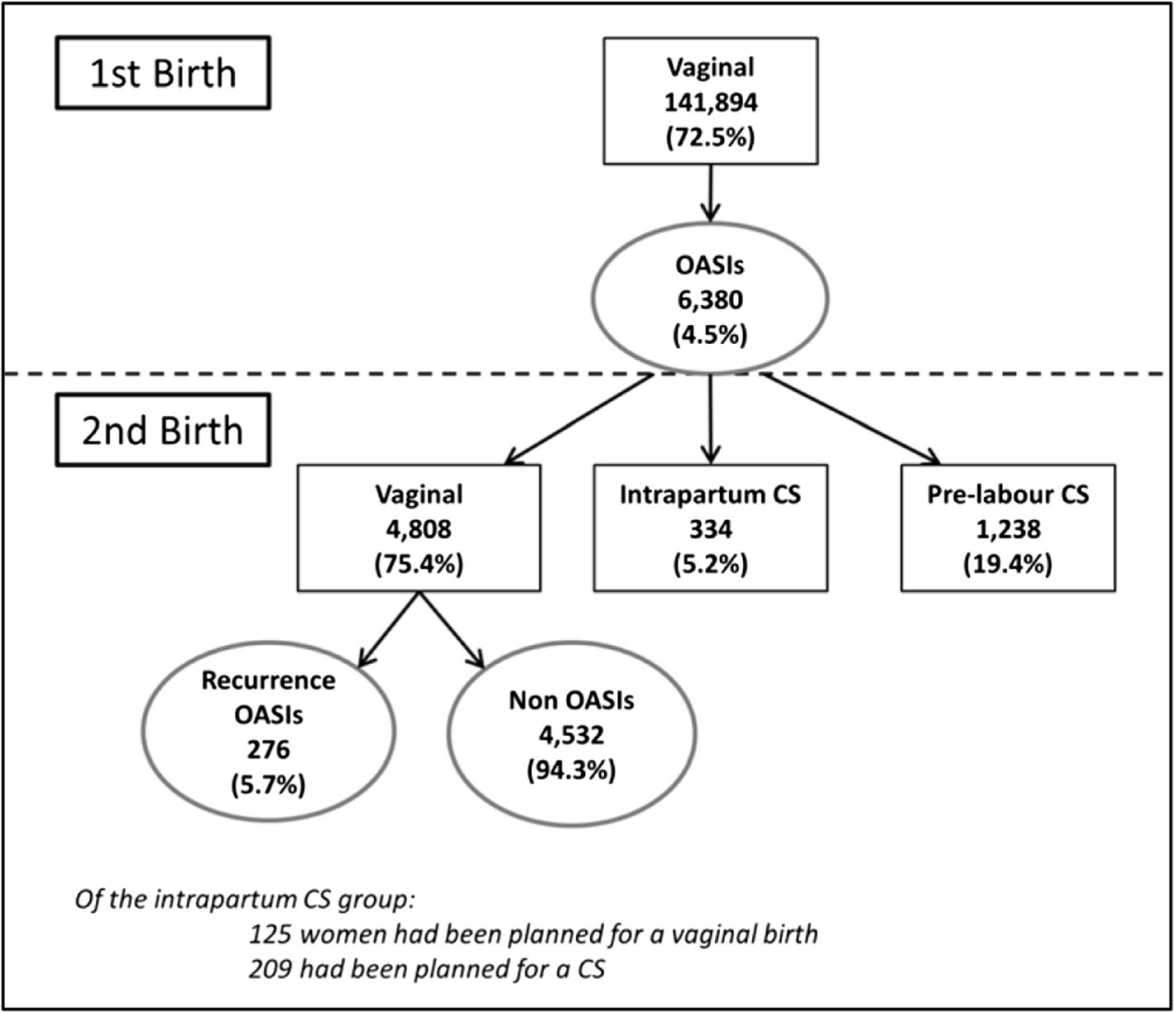
**OASI rates and mode of second birth among women with an OASI at first birth.**

Among 141,894 primiparous women in NSW 2001–2011 with a vaginal first birth and a subsequent second birth, 6,380 (4.5%) sustained an OASI. Of these women, 4,808 (75.4%) proceeded to a second vaginal birth, 1,238 (19.4%) to a pre-labour caesarean section, and 334 (5.2%) to an intrapartum caesarean. The OASI recurrence rate at second birth was 5.7% which was significantly higher than the first birth OASI rate (p < 0.01) (Figure 1).

### Association of first and second birth factors with OASI recurrence

Recurrence was investigated among the 4,808 women with a vaginal second birth following an OASI at first birth. Following exclusion of records with missing data, 4,773 (99.3%) records were available for analysis. After adjustment, the only factor from the first birth associated with an increased likelihood of OASI recurrence was diabetes (aOR 1.76 95%CI 1.03, 3.01), with a first born infant of ≥4.0 kg associated with decreased likelihood. Gestation at second birth of 37–38 weeks (compared with 39–40 weeks) was associated with a decreased odds of second OASI (aOR 0.55, 95% CI 0.36, 0.84), while birthweight ≥ 4.0 kg increased the likelihood of recurrence when compared with birthweights of 3.0- < 3.5 kg (aOR 2.89 95% CI 1.98, 4.22) as did birthweight of 3.5- < 4.0 kg (aOR 1.48 95% CI 1.08, 2.02) (Table 1). Analysis using a combined birthweight variable indicated that having a baby >4.0 kg at both births (aOR 1.63 95% CI 1.03, 2.57) or a baby <4.0 kg at first birth and a subsequent baby >4.0 kg (aOR 1.96 95% CI 1.39, 2.77) were associated with increased risk of recurrence compared to having two babies <4.0 kg (data not shown). While instrumental birth overall showed no association with recurrence, a sensitivity analysis indicated that forceps at the second birth carried an association with OASI recurrence compared with a non-instrumental birth (aOR 2.45, 95% CI 1.08, 5.55) while vacuum extraction did not (aOR 1.06 95% CI 0.59, 1.87). However, these results were based on very few births, with only 64 women having a forceps birth (of which 8 sustained an OASI).

**Table 1:** **Factors associated with OASI recurrence at second vaginal birth, 2001–2011**

			**OASI at 2nd birth**	**No OASI at 2nd birth**	**p**	**Cr ORs**	**Adj ORs with all risk factors in model**
			**n = 276**	**n = 4532**			
			**(5.7%)**	**(94.3%)**			
Country of birth*		Asian	47 (17.0)	910 (20.2)	0.23	0.82 [0.60, 1.13]	0.89 (0.63, 1.26)
		Non Asian	226 (81.9)	3594 (79.3)		Reference	Reference
**FIRST BIRTH FACTORS**							
	**Diabetes**	Yes	17 (6.2)	174 (3.8)	0.06	1.64 [0.98, 2.75]	1.76 [1.03, 3.01]
		No	259 (39.8)	4358 (96.2)		Reference	Reference
	**Epidural/spinal/general**	Yes	109 (39.5)	1993 (44.0)	0.14	0.82 [0.64, 1.05]	0.79 [0.59, 1.06]
		No	167 (60.5)	2539 (56.0)		Reference	Reference
	**Birth mode**	Instrumental	120 (43.5)	2007 (44.3)	0.79	0.97 (0.76, 1.24)	1.04 [0.78, 1.40]
		Non Instrumental	156 (56.5)	2525 (55.7)		Reference	Reference
	**Episiotomy**	Yes	121 (43.8)	1939 (42.8)	0.73	1.04 [0.82, 1.33]	1.13 [0.86, 1.50]
		No	155 (56.2)	2593 (57.2)		Reference	Reference
	**Birthweight(kg)**	<3.0	29 (10.5)	462 (10.2)	0.34	1.08 [0.70, 1.66]	1.40 [0.89, 2.19]
		3.0 - <3.5	95 (34.4)	1636 (36.1)		Reference	Reference
		3.5 - <4.0	119 (43.1)	1749 (38.6)		1.17 [0.89, 1.55]	0.94 [0.70, 1.27]
		≥ 4.0	33 (12.0)	685 (15.1)		0.83 [0.55, 1.25]	0.53 [0.34, 0.82]
Inter pregnancy interval (years)*		<1	50 (18.1)	1020 (22.5)	0.39	0.76 [0.52, 1.13]	0.78 [0.52, 1.17)
		1- < 2	126 (45.7)	1926 (42.5)		1.02 [0.74, 1.40]	1.04 [0.75, 1.45)
		2- < 3	59 (21.4)	919 (20.3)		Reference	Reference
		≥3	41 (14.9)	666 (14.7)		0.96 [0.64, 1.45]	1.01 [0.66, 1.54]
**SECOND BIRTH FACTORS**							
	**Mat age**	<25	29 (10.5)	550 (12.1)	0.61	0.87 [0.58, 1.30]	0.93 [0.61, 1.41]
		25-34	189 (68.5)	3110 (68.6)		Reference	Reference
		≥35	58 (21.0)	872 (19.2)		1.09 [0.81, 1.48]	1.07 [0.78, 1.46]
	**Hypertension**	Yes	16 (5.8)	161 (3.5)	0.05	1.67 [0.99, 2.84]	1.56 [0.90, 2.71]
		No	260 (94.2)	4371 (96.5)		Reference	Reference
	Gestation (weeks)*	<37	4 (1.5)	155 (3.4)	<0.01	0.36 [0.13, 0.99]	0.73 [0.24, 2.19]
		37-38	29 (10.5)	840 (18.5)		0.49 [0.33, 0.73]	0.55 [0.36, 0.84]
		39-40	197 (71.4)	2779 (61.3)		Reference	Reference
		≥41	46 (16.7)	757 (16.7)		0.86 [0.62, 1.19]	0.72 [0.51, 1.02]
	**Induction/Augmentation**	Yes	143 (51.8)	2428 (53.6)	0.57	0.93 [0.73,1.19]	0.95 [0.73, 1.24]
		No	133 (48.2)	2104 (46.3)		Reference	Reference
	**Epidural**	Yes	48 (17.4)	892 (19.7)	0.35	0.86 [0.62, 1.18]	0.95 [0.66, 1.38]
		No	228 (82.6)	3640 (80.3)		Reference	Reference
	**Birth mode**	Instrumental	24 (8.7)	289 (6.4)	0.13	1.40 (0.91, 2.16)	1.30 [0.80, 2.12]
		Non-instrumental	252 (91.3)	4243 (93.6)		Reference	Reference
	**Episiotomy**	Yes	68 (24.6)	1180 (26.0)	0.61	0.93 [0.70, 1.23]	0.84 [0.62, 1.14]
		No	208 (75.4)	3352 (74.0)		Reference	Reference
	Infant sex*	Male	158 (57.3)	2323 (51.3)	0.05	1.27 [1.00, 1.63]	1.12 [0.87, 1.44]
		Female	118 (42.7)	2208 (48.7)		Reference	Reference
	Birthweight(kg)*	<3.0	14 (5.1)	543 (12.0)	<0.01	0.54 [0.30, 0.96]	0.56 [0.23, 1.06]
		3.0 - <3.5	80 (29.0)	1675 (37.0)		Reference	Reference
		3.5 - <4.0	107 (38.8)	1653 (36.5)		1.36 [1.01, 1.83]	1.48 [1.08, 2.02]
		≥4.0	75 (27.2)	658 (14.5)		2.39 [1.72, 3.31]	2.89 [1.98, 4.22]
	**Year of second birth**	01-05	68 (24.6)	1098 (24.2)	0.24	Reference	Reference
		06-08	87 (31.5)	1643 (36.3)		0.86 [0.62, 1.19]	0.84 [0.60, 1.17]
		09-11	121 (43.8)	1791 (39.5)		1.09 [0.80, 1.48]	1.07 [0.78, 1.47]

*Missing data <0.7%, these categories may not total 100%.

Adjusted for all variables presented in table.

### Association of first birth with planned caesarean section for subsequent birth

Among the 6,380 women who had an OASI at their first birth, 1,447 (22.7%) were planned for a caesarean for their subsequent birth. Among those planned for a vaginal birth, 125 (2.5%) had a caesarean section. After exclusion of records with missing data, 6,337 (99.3%) records were available for analysis. Following adjustment, the following first birth factors remained associated with planned Caesarean: non-Asian country of birth; gestation ≥41 weeks; epidural, spinal or general anaesthetic; a fourth degree tear; birthweight ≥3.5 kg. Gestation at second birth <39 weeks and year of birth 2009–2011 were also predictive, with maternal age <25 years associated with a decreased likelihood of a planned caesarean section (Table 2). Excluding planned caesareans sections for non-vertex babies and placental conditions did not change estimates.

**Table 2:** **Association of first and second birth factors with planned caesarean section at second birth following OASI at first birth**

		**Planned CS**	**Planned vaginal**	**p**	**crOR**	**aOR**
		**n = 1447**	**n = 4933**			
		**(22.7%)**	**(77.3%)**			
Country of birth*	Asian	202 (14.0)	979 (19.9)	<0.01	0.66 (0.56, 0.77)	0.69 (0.58, 0.84)
	Non Asian	1236 (85.4)	3923 (79.5)			Reference
**FIRST BIRTH FACTORS**						
**Diabetes**	Yes	70 (4.8)	200 (4.1)	0.19	1.20 (0.91, 1.59)	1.17 (0.84, 1.63)
	No	1377 (95.2)	4733 (95.9)			Reference
**Hypertension**	Yes	133 (9.2)	430 (8.7)	0.58	1.06 (0.86, 1.30)	1.00 (0.79, 1.27)
	No	1314 (90.8)	4503 (91.3)			Reference
Gestation* (weeks)	<37	34 (2.4)	84 (1.7)	<0.01	1.49 (0.99, 2.23)	1.39 (0.87, 2.22)
	37-38	160 (11.1)	621 (12.6)		0.95 (0.78, 1.15)	0.81 (0.65, 0.99)
	39-40	782 (54.0)	2870 (58.2)		Reference	Reference
	≥41	471 (32.5)	1357 (27.5)		1.27 (1.12, 1.45)	1.42 (1.22, 1.65)
**Induction/augmentation**	Yes	1064 (73.5)	3352 (68.0)	<0.01	1.31 (1.15, 1.49)	1.11 (0.96, 1.30)
	No	383 (26.5)	1581 (32.0)			Reference
**Epidural/spinal or GA**	Yes	915 (63.2)	2185 (44.3)	<0.01	2.16 (1.92, 2.44)	1.73 (1.50, 2.00)
	No	532 (36.8)	2748 (55.7)			Reference
**Birth mode**	Instrumental	815 (56.3)	2209 (44.8)	<0.01	1.59 (1.41, 1.79)	1.16 (0.99, 1.35)
	Non-instr	632 (43.7)	2724 (55.2)			Reference
**Episiotomy**	Yes	718 (49.6)	2121 (43.0)	<0.01	1.31 (1.16, 1.47)	1.12 (0.98, 1.29)
	No	729 (50.4)	2812 (57.0)			Reference
**OASIS**	Fourth	267 (18.5)	203 (4.1)	<0.01	5.27 (4.35, 6.70)	4.95 (4.00, 6.13)
	Third/Unknown**	1180 (81.5)	4730 (95.9)			Reference
Birthweight (kg)*	<3.0	130 (9.0)	504 (10.2)	<0.01	1.10 (0.88, 1.37)	0.99 (0.77, 1.27)
	3.0- < 3.5	417 (28.8)	1772 (35.9)		Reference	Reference
	3.5- < 4.0	562 (38.8)	1918 (38.9)		1.25 (1.08, 1.44)	1.22 (1.04, 1.43)
	≥4.0	337 (23.3)	739 (15.0)		1.94 (1.64, 2.29)	1.66 (1.38, 2.01)
**Interpregnancy interval**	<1	291 (20.1)	1094 (22.2)	0.04	0.91 (0.76, 1.09)	0.95 (0.77, 1.17)
**(years)**	1- < 2	601 (41.5)	2099 (42.6)		0.98 (0.84, 1.15)	1.03 (0.86, 1.22)
	2- < 3	292 (20.2)	999 (20.3)		Reference	Reference
	≥3	263 (18.2)	740 (15.0)		1.22 (1.00, 1.47)	1.13 (0.91, 1.40)
**SECOND PREGNANCY**						
**Maternal age**	<25	136 (9.4)	593 (12.0)	<0.01	0.80 (0.65, 0.97)	0.75 (0.60, 0.93)
	25-34	972 (67.2)	3381 (68.5)		Reference	Reference
	35-40	309 (21.4)	897 (18.2)		1.20 (1.03, 1.39)	1.13 (0.96, 1.33)
	>40	30 (2.1)	62 (1.3)		1.68 (1.08, 2.62)	1.42 (0.87, 2.31)
**Diabetes**	Yes	93 (6.4)	269 (5.5)	0.16	1.19 (0.93, 1.52)	0.99 (0.74, 1.33)
	No	1354 (93.6)	4664 (94.5			Reference
**Hypertension**	Yes	53 (3.7)	187 (3.8)	0.82	0.97 (0.71, 1.32)	0.78 (0.55, 1.12)
	No	1394 (96.3)	4746 (96.2)			Reference
Gestation* (weeks)	<37	85 (5.9)	168 (3.4)	<0.01	2.03 (1.55, 2.67)	2.20 (1.64, 2.95)
	37-38	581 (4.02)	888 (18.0)		2.63 (2.31, 3.0)	2.87 (2.49, 3.31)
	39-40	757 (52.3)	3041 (61.7)		Reference	Reference
	≥41	24 (1.7)	835 (16.9)		0.12 (0.08, 0.18)	0.11 (0.07, 0.16)
**Year of second birth**	01-05	309 (21.4)	1194 (24.2)	0.03	Reference	Reference
	06-08	515 (35.6)	1774 (36.0)		1.12 (0.96, 1.32)	1.06 (0.89, 1.27)
	09-11	623 (43.0)	1965 (39.8)		1.22 (1.05, 1.43)	1.19 (1.01, 1.42)

*Missing data <0.7%, these categories may not total 100%.

**Third/Unknown contains 107 records for which OASI was ascertained by procedure coding for 3rd/4th degree tear, and hence degree (3rd or 4th) of tearing is unknown.

Adjusted for all variables presented in table.

## Discussion

Among women with a first and a second birth, we found a first birth vaginal OASI rate of 4.5% and an OASI recurrence rate of 5.7%. This recurrence rate sits within the range of 2.0% to 13.4% found in other studies [7,11-18,34-37]; with the majority, but not all [14,15,38] reporting increased risk for recurrence. Some of these differences may be associated with population characteristics, or related to different methods of ascertainment. Birth data has been shown to be less reliable than hospital data for correct identification of OASI [25], with under-ascertainment of OASI likely to under-estimate recurrence. Large population based cohort studies similar to ours demonstrate increases from first to second birth of 2.4% to 5.2% [13], 4.6% to 7.1% [16] and 1.9% to 3.7% [17]; and although the relative increases in risk are larger than that for our population, they are from lower first birth OASI rates.

Recurrence rates for other maternal conditions show higher relative increases in risk from one pregnancy to the next, for example, increase in postpartum haemorrhage rates from 5.8% at first birth to 14.8% at the second [39], pre-eclampsia rates from 4.1% to 14.7% [40], and breech presentations from 4.2% to 9.9% [41]. The lower relative increase for OASI recurrence rate compared to other conditions may be explained by altered management following a first birth with OASI. Given that 25% of births subsequent to a first birth OASI were by caesarean section, the pool of women at high risk for subsequent perineal trauma may have been reduced, with an unequal distribution of potential risk factors among women progressing to a second vaginal versus caesarean birth.

Investigation into first birth factors for recurrence has rarely been undertaken. We are aware of only one study which examined the role of a first birth factors with OASI recurrence [36]. The authors reported that a decrease in birthweight from first to second confers some degree of protection. Our analysis demonstrated that a heavier first baby was associated with a decreased likelihood of an OASI recurrence. Having experienced a first OASI with the birth of a small baby may be a proxy for other factors that we cannot identify in these data; for example, it may be associated with anatomical susceptibility, and is important when considering which women may be at risk of a second OASI. It may also be related to the unique population of women who have had a second vaginal birth following an OASI. Our finding of no association between OASI recurrence and a first birth episiotomy was in agreement with the previous study [36]. We believe our finding of an association of first birth diabetes with recurrence has not been previously identified.

Our result of increased OASI recurrence risk with higher birthweight at the second birth is consistent with other studies [11,13,15-17,36]. Women with a small baby experiencing an OASI at first birth whose next baby was >4 kg were particularly at increased risk of recurrence. While instrumental second births overall were not associated with recurrence, it is of note that similar to the findings of other studies [13,15,17,36], forceps did pose an increased risk however small numbers precluded separate analysis.

The decision around mode of delivery for second birth is challenging. Not only is risk of recurrence a consideration, but also whether a subsequent vaginal birth will worsen any symptoms or produce new problems, even in the absence of an OASI recurrence [42]. Our study has shown planned caesareans were more likely than planned vaginal births among women who were not Asian, if their first births were at gestation greater than 40 weeks, with regional or general anaesthetic, if their first-born infants were greater than 4 kg, or if they had a fourth degree tear at first birth. Why some of these factors were associated with planned caesareans is open to speculation, as these decisions likely include both medical and personal preferences. This information is not available in population data. Perhaps the presence of regional or general anaesthesia was associated with a difficult labour which women did not want to repeat, or was indicative of the repair required. With high birthweight a known risk factor for OASI, it is reasonable to assume that a caesarean would be planned with expectation of another large baby.

The presence of ongoing symptoms following OASI are likely to have had an effect on decision-making as well. The results of a recent questionnaire completed by UK consultant obstetricians reported that they would be far more likely to advise women to have a caesarean section if they had ongoing symptoms (including incontinence of flatus or stool) following a first OASI than those without symptoms [43]. This is in agreement with the Royal College of Obstetricians and Gynaecologists Guideline [44]. Whether OASI recurrence is more likely among women with symptoms than those who are symptom free is not known, as most studies to date have not examined this association but have focussed their outcomes on anal function and incontinence symptoms rather than recurrence per se [45]. While one small study concluded that ‘antepartum assessment…did not prove useful in identifying women in whom a further third degree was likely to occur’, it was based on only two cases of recurrence and is therefore not generalizable ([46] p151). With the relatively small increase between first occurrence and recurrence rates of 1.2% in the NSW population, women with predisposing risk factors for recurrence may in fact have delivered by caesarean at their subsequent birth. The lower recurrence rate reported in our population compared to other studies may relate to different decisions made about mode of delivery at second birth. As such the recurrence rate of 5.7% may not reflect the true risk of recurrence for all women who have had a first OASI.

The results of this study demonstrate the complexity of counselling a woman approaching a second birth following a first birth OASI, when the option of a caesarean section may be being discussed. If a woman does progress to a second labour and vaginal birth, and requires an induction or augmentation, an epidural, episiotomy or vacuum birth, the results from this large population study provide some reassurance that there is no increased risk for OASI recurrence. In addition, with only a small increase in OASI rates from one birth to the next and with a paucity of risk factors identified for recurrence, it appears that women who were at higher risk were appropriately being delivered by caesarean section.

### Strengths and limitations of this study

Longitudinal linkage of these large population-based datasets allowed exploration of the association of first and second birth factors and obstetric interventions with a subsequent adverse outcome. Our data allowed us to identify consecutive births, and exclude the small number of women who birthed outside NSW for either their first or second birth. Records that were excluded due to missing data were small in number (less than 1%), and the variables we used for analysis have previously been validated as reliably and accurately reported [25,29-31]. Ethnicity could only be determined by self-reported country of birth. The analysis was limited by having no information available in population data on the influence of symptoms, personal preference or other factors that may have affected clinical decision-making for the second birth mode. There is also possibility of increased vigilance in detection and reporting of a subsequent adverse outcome. We have demonstrated in another study that recorded history of postpartum haemorrhage was associated with an increased risk of reporting a recurrent event, however recurrence rates approximated the true recurrence [47].

## Conclusion

We have demonstrated that for women who proceed to a subsequent vaginal birth the recurrence risk of OASI is approximately 1 in 20, and not markedly higher than the risk for an OASI at first birth. Significant risk factors for OASI recurrence included diabetes recorded at first birth and large infant birthweight at second birth. The profile for women having a planned caesarean section differed from those who planned to deliver vaginally, which likely influenced the OASI recurrence rate.
